# A novel resource for genomics of Triticeae: BAC library specific for the short arm of rye (*Secale cereale *L.) chromosome 1R (1RS)

**DOI:** 10.1186/1471-2164-9-237

**Published:** 2008-05-21

**Authors:** Hana Šimková, Jan Šafář, Pavla Suchánková, Pavlína Kovářová, Jan Bartoš, Marie Kubaláková, Jaroslav Janda, Jarmila Číhalíková, Rohit Mago, Tamas Lelley, Jaroslav Doležel

**Affiliations:** 1 Laboratory of Molecular Cytogenetics and Cytometry, Institute of Experimental Botany, Sokolovská 6, CZ-77200 Olomouc, Czech Republic; 2 Department of Cell Biology and Genetics, Palacký University, Šlechtitelů 11, CZ-78371 Olomouc, Czech Republic; 3 CSIRO Plant Industry, GPO box 1600, Canberra, ACT 2601, Australia; 4 Department of Agrobiotechnology, IFA-Tulln, Konrad Lorenz Str. 20, A-3400 Tulln, University of Natural Resources and Life Sciences, Vienna, Austria

## Abstract

**Background:**

Genomics of rye (*Secale cereale *L.) is impeded by its large nuclear genome (1C~7,900 Mbp) with prevalence of DNA repeats (> 90%). An attractive possibility is to dissect the genome to small parts after flow sorting particular chromosomes and chromosome arms. To test this approach, we have chosen 1RS chromosome arm, which represents only 5.6% of the total rye genome. The 1RS arm is an attractive target as it carries many important genes and because it became part of the wheat gene pool as the 1BL.1RS translocation.

**Results:**

We demonstrate that it is possible to sort 1RS arm from wheat-rye ditelosomic addition line. Using this approach, we isolated over 10 million of 1RS arms using flow sorting and used their DNA to construct a 1RS-specific BAC library, which comprises 103,680 clones with average insert size of 73 kb. The library comprises two sublibraries constructed using *Hin*dIII and *Eco*RI and provides a deep coverage of about 14-fold of the 1RS arm (442 Mbp). We present preliminary results obtained during positional cloning of the stem rust resistance gene *SrR*, which confirm a potential of the library to speed up isolation of agronomically important genes by map-based cloning.

**Conclusion:**

We present a strategy that enables sorting short arms of several chromosomes of rye. Using flow-sorted chromosomes, we have constructed a deep coverage BAC library specific for the short arm of chromosome 1R (1RS). This is the first subgenomic BAC library available for rye and we demonstrate its potential for positional gene cloning. We expect that the library will facilitate development of a physical contig map of 1RS and comparative genomics of the homoeologous chromosome group 1 of wheat, barley and rye.

## Background

Since the beginning of the twentieth century, attempts were made to transfer useful genes from related species into cultivated wheat through interspecific hybridization. Following crosses between wheat and rye, in the first half of the twentieth century, several wheat varieties were developed; initially with the complete rye chromosome 1R substituted for wheat chromosome 1B, later substituting only the 1RS chromosome arm for the short arm of chromosome 1B in a form of the 1BL.1RS translocation [45]. Other translocations were developed later, involving wheat 1AL and 1DL chromosome arms instead of the 1BL [37,46]. These translocations conferred resistance to wheat against diseases, such as powdery mildew, leaf rust, stem rust, yellow rust, and against insects such as green bug and wheat curl mite, the latter being a vector of wheat streak mosaic virus [4,28,37,46]. The 1RS chromosome is also believed to improve the adaptation and increases yield of wheat in certain genetic backgrounds [2,21].

Due to its valuable features, the 1BL.1RS translocation was integrated into wheat breeding programs all over the world, and more than 300 wheat cultivars were known to carry it at the end of the 20^th ^century [32]. Cultivars with 1RS occupy now a considerable acreage of the wheat growing area as compared to other wheat-alien translocations and the 1RS chromosome arm is by far the most widely used alien chromatin in wheat breeding [10,12]. In fact, 1RS is now part of the wheat gene pool and it would be difficult to eliminate it. A better knowledge of 1RS at the molecular level is needed to clone genes underlying the resistance phenotypes, support further improvement of existing wheat cultivars, and eliminate some negative effects of the translocation on bread making quality [15,23,33].

1RS translocations from at least two different rye genotypes carry stem rust resistance genes. The *Sr31 *gene from rye cv. 'Petkus' has been used widely in CIMMYT-derived wheat cultivars to prevent wheat stem rust infection. However, the recent spread of a new stem rust pathotype Ug99 [31], which is virulent on *Sr31*, caused concern among wheat growers worldwide because in many current wheat varieties, this gene provides the only protection against the disease. Until now, the stem rust resistance gene *SrR*, which is located on 1RS of cv. 'Imperial', has not been extensively used. Tests done at University of Minnesota have shown that *SrR *is effective against Ug99 (Jim Kolmer, Yue Jin and R. Mago, unpublished) and so this gene could provide a useful component of stem rust resistance gene pyramids in new wheat varieties resistant to Ug99 and, at the same time, potentially provide the additional positive yield and adaptation features of 1RS from cv. 'Petkus'.

We have previously shown that the region carrying *SrR *on 1RS of cv. 'Imperial' has synteny with the region on 1HS of barley, which carries the powdery mildew resistance gene *Mla *[25]. Stem rust susceptible mutants obtained in a wheat 1BL.1RS translocation stock indicated that *Mla *might be an ortholog of the stem rust resistance gene *SrR *[26]. Hybridization with a probe for barley *Mla1 *gene identified ~20 copies on 1RS of 'Imperial' rye. In some interstitial deletion mutants obtained by gamma irradiation, only three of these 20 members were lost. Thus indicating that these three deleted copies may be candidates for the stem rust resistance gene *SrR*.

The analysis of the rye genome at the molecular level is hampered by its size (1C~7900 Mbp) [6], which is about 1.4-fold larger than the genomes of diploid progenitors of bread wheat. Although proportion of repeated DNA in the rye genome is not known, considering recent estimates in wheat [30], repetitive DNA may constitute at least 90% of the rye genome. The analysis at molecular level could be simplified by dissecting the genome to small parts and creating subgenomic molecular resources such as chromosome arm-specific BAC libraries. The short arm of 1R represents only 5.6 % of the whole genome [36] and hence only about 442 Mbp. The proposed approach would reduce the amount of genetic information to be studied by 18-fold and facilitate targeted investigation of this important part of the rye (and wheat) genome.

We have shown previously that flow cytometry can be used to sort particular chromosomes in a range of plant species, including wheat [20,43], barley [24,41] and rye [18]. DNA of flow-sorted chromosomes has been used to construct subgenomic BAC libraries in hexaploid wheat. Until now, a composite BAC library specific for 'Chinese Spring' chromosomes 1D, 4D and 6D was created [13], as well as BAC libraries from chromosome 3B of 'Chinese Spring' [35] and 3B of 'Hope' (in preparation), and libraries specific for hexaploid wheat chromosome arms 1BS of 'Pavon' [14], 3AS of 'Chinese Spring' [39] and 3DS of 'Chinese Spring' (in preparation).

Here we show for the first time that it is possible to sort the arm of a rye chromosome using flow cytometry. The short arm of chromosome 1R (1RS) was sorted from a 1RS wheat-rye ditelosomic addition line and used to construct the first subgenomic BAC library for rye, representing only 5.6% of the genome. The library was constructed using two restriction enzymes and provides a deep coverage of 1RS. We demonstrate its potential to speed up positional cloning of agronomically important genes such as the *SrR *gene by map-based cloning.

## Results

**Figure 1:**
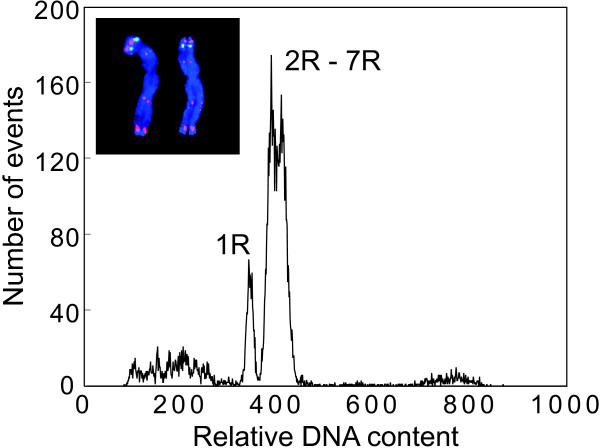
**Histogram of relative fluorescence intensity ('flow karyotype') obtained after flow cytometric analysis of DAPI-stained chromosome suspension of *Secale cereale *'Imperial'**. The flow karyotype consists of a composite peak representing chromosomes 2R – 7R, and a peak representing chromosome 1R. Insert: Images of flow-sorted chromosome 1R after FISH with probes for 5S rDNA (green color) and *pSc*119 (red color) DNA sequences. The chromosomes were counterstained with DAPI (blue color).

Flow cytometric analysis of mitotic metaphase chromosomes isolated from rye cv. 'Imperial' resulted in a histogram of relative fluorescence intensities (flow karyotypes) with a composite peak representing chromosomes 2R – 7R and a small peak of chromosome 1R (Figure 1). This is the smallest chromosome in the karyotype and the only one that could be sorted individually. The chromosome is characterized by a secondary constriction separating the satellite from the short arm and the 5S rDNA locus on the satellite, and its identity in the sorted fraction was confirmed by FISH with probes for 5S rDNA and pSc119.2 (Figure 1, insert).

**Figure 2:**
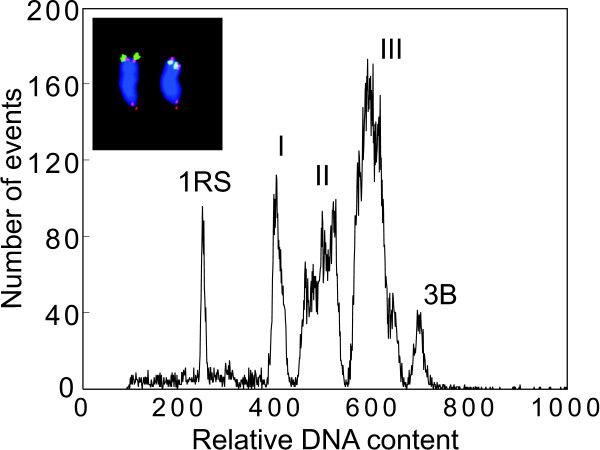
**The flow karyotype of the wheat-rye 1RS telosome addition line**. The karyotype contains four peaks representing the chromosomes of wheat (labeled I, II, III, and 3B) and a peak of the telocentric chromosome 1RS. Insert: Images of the flow-sorted chromosome 1RS after FISH with probes for telomeric sequences (red color) and *pSc*200 (green color) DNA sequences. The chromosomes were counterstained with DAPI (blue color).

Although chromosome 1R could easily be sorted, we considered 1,000 Mbp still too large fraction of the rye genome (~12.7%). Therefore, to simplify the analysis of 1RS, we targeted only this particular region and tested the suitability of 1RS wheat-rye ditelosomic addition line for sorting 1RS. Flow karyotype obtained from this line was complex and consisted of four peaks representing various wheat chromosomes and a peak of telocentric chromosome 1RS (Figure 2). The telosome could be sorted and the identity of sorted 1RS was determined by FISH with probes for pSc200 and telomeric repeats (Figure 2, insert). The contamination of sorted 1RS fractions ranged from 5 to 15% depending on the speed of analysis, with low speeds (below 500 particles/sec) resulting in higher resolution of the 1RS peak and higher purities. The contamination was not due to specific chromosomes, and various wheat chromosomes and their fragments were observed in the sorted fraction of 1RS.

In order to produce enough seeds for the 1RS BAC library construction, seeds from cytologically controlled 1RS ditelosomic wheat-rye addition plants were used. Whereas monotelosomic plants produce only ca. 40% monotelosomic progeny, with the remaining plants not containing any 1RS chromosome, the progeny of ditelosomic plants are up to 60% ditelosomic, with the remaining plants being monotelosomic. Only the use of such material permitted preparation of chromosome suspensions with sufficient concentration of chromosomes to achieve good resolution of the 1RS arm on a flow karyotype and high yields during flow sorting.

In total, 10.3 × 10^6 ^1RS chromosome arms were sorted from 540 samples that were prepared from 11,600 seeds. To avoid excessive sorting times, the samples were run at ~1000 particles/sec and the average sort rate was 25 chromosomes/sec. The sorting took 60 working days and the average purity in the sorted fractions estimated by FISH using probes for 45S rDNA and pSc200 was 86%. The first batch of 5.1 × 10^6 ^chromosomes were embedded in 30 agarose miniplugs, each containing 1 × 10^5 ^or 2 × 10^5 ^chromosomes, and used for construction of a *Hin*dIII library. This number of chromosomes corresponded to approximately 4.6 μg of DNA. For a *Bam*HI library, 5.2 × 10^6 ^chromosomes were sorted in batches of 4 × 10^5 ^and embedded in 13 agarose miniplugs. This amount represented about 4.7 μg of DNA.

**Figure 3:**
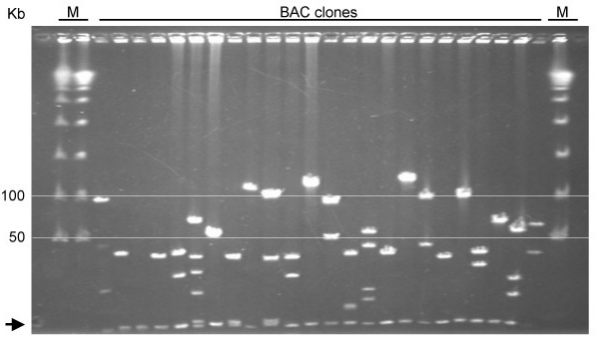
**Analysis of inserts in the 1RS BAC library**. 24 randomly selected BAC clones were digested with *Not*I, run on PFGE and stained with ethidium bromide. M = molecular size marker Lambda Ladder. The arrow indicates the vector.

**Figure 4:**
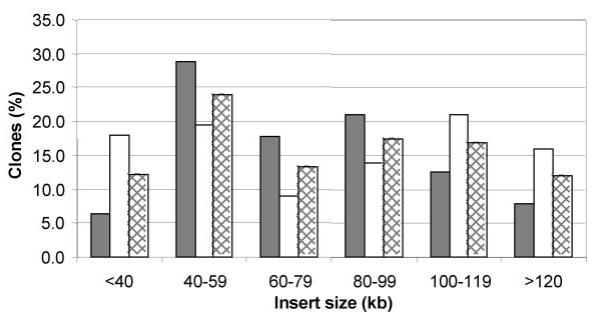
**Insert size distribution in the 1RS BAC library**. 191 random clones from the *Hin*dIII sub-library (SccImp1RShA) and 201 random clones from the *Bam*HI sub-library (SccImp1RSbA) were isolated and sized using PFGE. Grey bars: *Hin*dIII sub-library; white bars: *Bam*HI sub-library; hatched bars: Complete 1RS BAC library.

Chromosomal DNA partially digested either with *Hin*dIII or *Bam*HI was then used to construct two 1RS-specific BAC libraries. The first BAC library (SccImp1RShA) constructed using *Hin*dIII consisted of 66,816 clones obtained from four size fractions of DNA: H01 (constructed from the 50–75 kb fraction) consisted of 8,064 clones, H02 (75–120 kb) contained 31,488 clones, H1 (120–170 kb) was represented by 15,360 clones and H2 (170–300 kb) by 11,904 clones. As the insert size did not differ significantly among the particular fractions (data not shown), the average insert size was estimated for the complete *Hin*dIII library (Figure 3). Based on analysis of 191 clones, the mean insert size of this library was found to be 72 kb (Figure 4) with a median at 70 kb. The smallest insert was 15 kb while the largest was 160 kb. As the purity of the sorted fraction estimated by FISH showed to be 86% and the percentage of empty clones was 6%, the *Hin*dIII library was estimated to cover the 1RS chromosome arm 8.8-fold.

The second library (SccImp1RSbA) was constructed from *Bam*HI-digested DNA and comprised 36,864 clones obtained from three size fractions: C2 (75–115 kb), C3 (115–175 kb) and C4 (175–300 kb). Similarly to the *Hin*dIII library, the insert sizes resembled each other in all three fractions thus they were not analyzed separately. Based on analysis of 201 clones the observed mean insert size reached 75 kb with a median at 80 kb (Figure 4). The smallest insert was 15 kb while the largest was 160 kb. Considering the purity of the sorted fraction of 86% and 3% empty clones, the coverage of the *Bam*HI library was calculated to be 5.2-fold.

The complete 1RS-specific BAC library consisted of 103,680 clones ordered in 270 × 384-well microtitre plates. Almost one third of the 1RS library (30%) had inserts larger than 100 kb, while 26% inserts were distributed from 70 to 100 kb, and 44% of inserts were shorter than 70 kb. With the average insert size of 73 kb, the library represents 14 equivalents of the 1RS chromosome arm. Using the formula of Clarke and Carbon [3], the probability of finding any sequence from the short arm of chromosome 1R in the library was 99.9%.

The chromosome arm specificity and predicted coverage of the 1RS arm, was confirmed by screening the BAC library by PCR with a set of molecular markers. This was done on 270 DNA pools, each of them prepared from one 384-well plate. The pools were screened with one SSR marker and five STS markers specific for the short arm of chromosome 1R. The number of pools that gave PCR products of the expected lengths with particular markers ranged from 6 to 14 (Table 1). Based on these results, we estimated the coverage of the complete 1RS BAC library to be at least 11-fold.

**Table 1:** Results of 1RS BAC library screening on pools of 384-well plates with the 1RS-specific DNA markers

**Marker code**	**Reference**	**Primers**	**Number of positive pools**
SSR markers			
scm9	Saal and Wricke 1999	TGACAACCCCCTTTCCCTCGTTCATCGACGCTAAGGAGGACCC	10

STS markers*			
bcd1434 ^1^	Loarce et al. 1996	CCACCTTCTCGCTGTTGAAT CTGCTGTCCAGCCAGAAAAT	11
mwg2062 ^2^	Korzun et al. 1998	TCTCGCTGGTATTCAGGGTCC AAACGATAGCAAGAGGAACCG	11
psr109 ^3^	Masojc et al. 2001	GACTACCTGATCCGCTCCAA ACTTGCACTTTAGGCCTTGC	6
psr949 ^4^	Masojc et al. 2001	GCCCTGCCATATAACTTCCA GCATCATGTCTCCCTTTACCA	16
IB-262	Mago et al. 2002	GTAGGTAATGTATCAGAGTTGTAC GTCTTTGTGCTCGGTAGCTCC	14
Average			11.3

* developed from RFLP markers

^1 ^primers designed from bcd1434-3 sequence (GrainGenes )

^2 ^primer sequences downloaded from GrainGenes

^3 ^primers designed from psr109 sequence (GrainGenes )

^4 ^primers designed from psr949-Forward sequence (GrainGenes )

**Figure 5:**
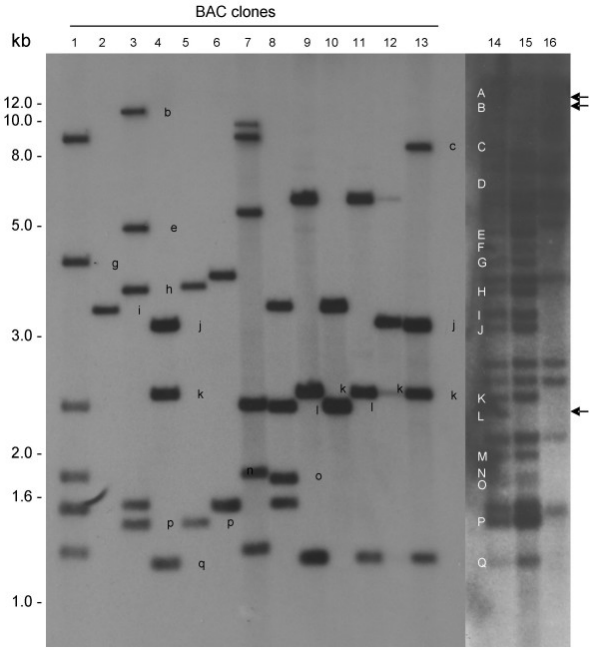
**Hybridization of the marker B76 with the *Dra*I-digested DNA of selected BAC clones and the wheat lines**. Lanes 1–13: 1RS BAC clones showing some of the rye fragments missing in deletion mutants. Lane 14: Genomic DNA of Gabo1BL.1RS (wildtype; carrying the 1RS from cv. 'Imperial'); lane 15: interstitial deletion mutant (00.002); lane 16: mutant with chromosome arm loss (00.001). The capital letters (A to Q) show the position of rye fragments orthologous to the barley *Mla *resistance gene. The corresponding BAC fragments are shown as small letters. Arrows indicate the position of fragments (A, B & L) missing in the interstitial deletion mutant and maybe candidate for the stem rust resistance gene *SrR*. A detailed picture of the mutants with the loss of 1RS fragments is shown in Mago *et al*. (2004). All the samples were electrophoresed in the same gel however, two autoradiograms were developed. A 30 min exposure for the BAC clones and an overnight exposure to see the corresponding bands in the genomic DNA.

To test the utility of the library for isolating candidates for the *SrR *gene, BAC filters from both *Bam*HI and *Hin*dIII sub-libraries were used for hybridization with the B76 probe, derived from the LRR region of the *Mla *gene [26] and a total of 165 positive clones were identified. Previously, using hybridization of wheat 1RS.1BL genomic DNA with the B76 probe, we identified ~20 *Mla*-orthologous *Dra*I-digested fragments on 1RS of rye cv. 'Imperial' [26] of these at least three were missing in interstitial deletion mutants. DNA from the 165 BAC clones was digested with *Dra*I to identify the BACs carrying these three fragments missing in the interstitial deletions. Figure 5 shows hybridization of the B76 probe to wheat genomic DNA and selected BACs digested with *Dra*I. At least three BAC clones (no. 3, 8 and 10) were confirmed to carry two of the fragments (B and L) missing in interstitial deletion mutants based on fragment size. This was also confirmed using a PCR marker developed from BACs no. 3 and 10 which amplified the expected product from the wildtype but not the deletion mutant indicating that the BAC is from the expected region (data not shown). In total 19 BAC clones were identified carrying the three fragments missing in the mutants.

## Discussion

This work focuses on the short arm of rye chromosome 1 (1RS), which represents an important part of not only the rye genome but also the genomes of many cultivars of wheat. However, while the 1RS arm contains several genes of agronomic importance such as disease resistance, yield enhancement, its genetic makeup has a negative impact on bread making quality. Since, rye chromosomes do not recombine regularly with wheat chromosomes, getting rid of the negative characters is not easy by conventional recombination and (standard genetic) segregation. It is hoped that advanced tools of genomics will assist in cloning useful genes from 1RS.

Flow cytometric analysis of chromosomes prepared from rye cv. 'Imperial' confirmed our previous observation [18] according to which only chromosome 1R can be discriminated and sorted from this cultivar. Moreover, until now we have not identified other rye cultivars from which chromosome 1R could be sorted. The ability to sort only one chromosome would greatly limit the application of flow cytogenetics in rye to dissect its nuclear genome. In order to overcome this limitation, we adopted the strategy based on using wheat-rye chromosome addition lines [18]. Since the rye chromosomes are larger than any of the wheat chromosomes, chromosomes 2R – 7R can be sorted from appropriate cytogenetic stocks. These chromosomes represent 13.2% – 16.6% of the rye genome [36].

This paper expands the potential of flow cytogenetics for genomics of rye by demonstrating a possibility to sort single chromosome arms. Our system is similar to that described above and to that used by Suchánková *et al*. [41] to sort chromosome arms of barley, and relies on using wheat-rye telosomic addition lines. We demonstrate that using this system, it is possible to purify the short arm of chromosome 1R (1RS). Considering the relative lengths of rye [36] and wheat [11] chromosomes and the genome sizes of these species [1], it may be predicted that, except 2RS, short arms of all rye chromosomes can be discriminated and sorted from respective wheat-rye telosome addition lines. However, it should be noted that only 3RS, 4RS, 5RS and 7RS wheat-rye telosomic addition lines are currently available [29] and that the 5RS line is unstable (B. Friebe, pers. comm.). On the other hand, peaks of long arms of most of rye chromosomes would overlap with the peak I representing wheat chromosomes 1D, 4D and 6D, and thus only 1RL and 3RL would be sortable.

Flow-sorted chromosomes were shown to be suitable in a number of applications in plant genome analysis and mapping, with the construction of chromosome (arm)-specific BAC libraries being the most attractive [7,8]. Šafář *et al*. [35] were the first to show that it was possible to construct BAC libraries from small amounts of high molecular weight DNA that can be prepared from flow-sorted chromosomes. Following this work, BAC libraries were prepared from other wheat chromosomes [[13,14], in preparation]. Chromosome-specific BAC libraries facilitate analysis of molecular structure of targeted genome regions, development of molecular markers from these regions and construction of physical contig maps [30] as well as positional gene cloning [17]. While six chromosome (arm)-specific BAC libraries have already been created in hexaploid wheat, until now this system has not been verified in other eukaryotic species.

This paper proves that the strategy of constructing BAC libraries from particular chromosome arms is applicable also for rye. Whereas the coverage of the 1RS arm by the whole library was estimated to be 14-fold considering insert size of BAC clones, library screening with a set of DNA markers indicated an 11-fold coverage. Over ten-fold coverage of 1RS was confirmed during the first phase of positional cloning of the *SrR *gene. All these estimates confirmed the deep coverage of the 1RS arm. The average insert size of the 1RS-specific library is similar to that observed for the 1D, 4D and 6D-specific library (85 kb), and BAC libraries specific for chromosome arms 1BS (82 kb) and 3AS (80 kb), but is lower than the average insert size of the 3B-specific library with 103 kb. As shown in Figure 4, the relatively low insert size is due to presence of a rather abundant class of smaller inserts (40 – 59 kb) representing 29% of the *Hin*dIII library and 19% of the *Bam*HI library. Our protocol for BAC library construction includes only one size selection step prior to cloning due to low amounts of high molecular weight DNA obtained from sorted chromosomes. However, we continue working on improving the protocol to allow for two-step size selection. Nevertheless, the 1RS library contains 56% clones with inserts over 70 kb. These clones represent 11-fold coverage of the 1RS arm (calculated using the insert size), which makes the library suitable for construction of a physical contig map.

The isolation of the BACs carrying the three *Mla *orthologs missing in the deletion mutants demonstrates the value of this library in constructing a DNA sequence contig from the 1RS region identified by deletion mutant analysis as the region containing *SrR*. It indicates that the 1RS library is an excellent resource for rye and wheat researchers looking at the genes encoding important agronomic traits associated with 1RS. For example, as shown in Figure 5, the 13 BACs carry at least 13 of the about 17 *Mla*-orthologous fragments identified on 1RS. Thus, the 165 BACs identified with B76 probe would indicate over ten-fold coverage of the region. Our previous attempts to isolate the same region of 1RS from ungridded BAC library from a wheat 1BL.1RS translocation line proved unsuccessful (unpublished). Further characterization of the BAC clones is in progress to confirm whether any of the candidates are stem rust *SrR *gene/s.

In addition to development of molecular markers and construction of physical map, the library may find other attractive uses. For example, it has already been used to study the molecular structure of rye chromosome ends [42]. Moreover, its availability provides the opportunity to perform detailed comparative analysis of molecular structure of wheat chromosome group 1 and rye chromosome 1R. In fact, 1R is the only rye chromosome, which is collinear along its length with the wheat chromosomes of homoeologous group 1 [5].

## Conclusion

Here we present a strategy that enables sorting of short arms of several chromosomes of rye. Using flow-sorted chromosomes, we have constructed a deep coverage BAC library specific for the short arm of chromosome 1R (1RS). The library is the first subgenomic BAC library available for rye and we demonstrate its potential for positional gene cloning. We expect that the library will facilitate the analysis of molecular structure of 1RS, evolution of Triticeae genomes, and cloning important genes located on this arm. The library is available for academic users by reimbursement of duplication costs. Academic and commercial users interested in a copy of the BAC library should contact the corresponding author.

## Methods

### Plant material

Seeds of rye (*Secale cereale *L., 2*n *= 2*x *= 14) cv. 'Imperial' were obtained from Prof. A. J. Lukaszewski (University of California, Riverside, USA). Original seeds of 1RS wheat-rye ditelosomic addition line, derived from rye cv. 'Imperial' in the background of wheat (*Triticum aestivum *L., *2n = 6x *= 42) cv. 'Chinese Spring' (Driscoll and Sears 1971), were provided by Dr. B. Friebe (Kansas State University, Manhattan, USA). For cell cycle synchronization, seeds from cytologically selected plants were germinated in the dark at 25 ± 0.5°C on moistened filter paper in glass Petri dishes for 2–3 days to achieve optimal root length (2–3 cm).

### Preparation of chromosome suspensions and sorting of chromosomes

Cell-cycle synchronization, accumulation of metaphases in root tips and preparation of chromosome suspensions were performed according to Kubaláková *et al*. (2003). Briefly, chromosome suspensions were prepared by mechanical homogenization of 30 formaldehyde-fixed root tips (~1 mm) in 1 ml ice-cold isolation buffer (IB, Šimková *et al*. 2003). Chromosomes in suspension were stained by 2 μg/ml DAPI (4',6-diamidino-2-phenylindole) and analyzed using a FACSVantage SE flow cytometer (Becton Dickinson, San José, USA). Sort windows were set on a dot plot of fluorescence pulse area versus fluorescence pulse width. The purity in sorted fractions was checked regularly by fluorescence *in situ *hybridization with chromosomes sorted onto a microscope slide. For BAC library construction, chromosome arm 1RS was sorted in aliquots of 1 × 10^5^, 2 × 10^5 ^and 4 × 10^5 ^copies into 160 μl, 320 μl and 640 μl 1.5 × IB, respectively.

### Fluorescence in situ hybridization (FISH)

2000 particles were flow-sorted from a peak assumed to represent 1RS into 15-μl drop of PRINS buffer (Kubaláková *et al*. 2001), which was supplemented with 5% sucrose, onto a microscope slide. After air-drying, flow-sorted chromosomes were identified using FISH with probes for 5S rDNA, 45S rDNA, pSc119.2 and pSc200 repeats, which provide chromosome-specific fluorescent banding patterns (Kubaláková *et al*. 2003). The chromosomes were counterstained with DAPI, and the preparations were evaluated using Olympus BX60 microscope (Olympus, Tokyo, Japan) with CCD camera interfaced to a PC running the ISIS software (Metasystems, Altlussheim, Germany).

### BAC library construction

Preparation of high-molecular-weight DNA and construction of BAC library from 1RS were performed as described by Šimková *et al*. (2003) and Šafář *et al*. (2004). Briefly, each batch of flow-sorted 1RS arm was pelleted at 200 or 400 *g *and mixed with low-melting-point agarose to form 15-μl agarose mini-plugs. Proteinase-treated DNA in the mini-plugs was partially digested either with *Hin*dIII or *Bam*HI and size-selected by pulsed field gel electrophoresis (PFGE, 6V/cm, switch time 1–40 s, for 12 h and switch time 2.5–5.5 s for 6 h at 14°C). Fragments of 50–300 kb were subdivided into smaller fractions and isolated from the agarose gel by electroelution. The size-selected DNA was ligated to dephosphorylated vector pIndigoBAC-5 (Epicentre, Madison, USA) in molar ratio of 1 to 5 (in BAC vector excess). Four ligations corresponding to fragment size of 50–75 kb (H01fraction), 75–120 kb (H02 fraction), 120–170 (H1 fraction) and 170–300 kb (H2 fraction), respectively, were prepared from *Hin*dIII-digested DNA and three ligations, corresponding to 75–115 kb (C2 fraction), 115–175 kb (C3 fraction) and 175–300 kb (C4 fraction), respectively, were prepared from the *Bam*HI-digested DNA. The ligation mixtures were desalted and used to transform *Escherichia coli *ElectroMAX DH10B competent cells (Gibco BRL, Gaithersburg, USA) by electroporation. The library was ordered in 384-well plates filled with freezing medium (Woo *et al*. 1994) and stored at -80°C.

### Characterization of BAC inserts

Three hundred and ninety two randomly selected BAC clones proportionally representing all size fractions from both libraries were used to estimate the average insert size. The DNA was isolated using standard alkaline lysis method and digested with *Not*I (0.4 U/20 μl). The obtained DNA fragments were separated in 1% agarose gel in 0.5 × TBE buffer by PFGE at 6V/cm, switch time ramp 1–40 sec, 14°C for 14 h. The size of the fragments was estimated by comparing with two size markers: Lambda Ladder PFG Marker and MidRange Marker I (New England Biolabs, Beverly, USA).

### Estimation of 1RS coverage using molecular markers

BAC clones were grown overnight in 384-well plates containing 2YT medium supplemented with chloramphenicol (12.5 μg/ml). Clones from each of the 384-well plates were pooled, pelleted and resuspended in 4 ml of TE (10 mM Tris, 1 mM EDTA) buffer. In order to prepare DNA pools, the bacterial suspensions were lysed at 95°C for 30 min, pelleted at 3,000 g for 60 min and the supernatant was diluted 25-fold with deionized water. PCR reaction mix (10 μl) consisted of 1 μl template DNA of diluted pool, 1× Buffer for DyNAzyme DNA Polymerase (Finnzymes, Espoo, Finland), 0.2 mM dNTPs, 1 μM primers (Table 1), and DyNAzyme II DNA Polymerase (Finnzymes). PCR reaction was performed as follows: denaturation for 4 min at 94°C followed by 30–35 cycles consisting of denaturation at 94°C for 30 s, annealing at appropriate temperature (58°–62°C) for 40 s, extension at 72°C for 40 s, and final extension at 72°C for 10 min. Presence of PCR products was checked by electrophoresis in 1% agarose gels run in 0.5 × TBE buffer.

### Screening of high-density filters and BAC DNA isolation

High-density colony filters were prepared by spotting BAC clones in duplicate in a 4 × 4 gridding pattern on Hybond N+ nylon membranes (Amersham Biosciences, Piscataway, USA) using the GeneTAC G3 robot (Genomic Solutions, Ann Arbor, USA). 18,432 individual colonies (36,864 spots in total) were arrayed on one filter. DNA probes for hybridization to screen the BAC library or blotted DNA digests were labeled with [^32^P]dCTP using Megaprime DNA Labeling System (Amersham). The probes derived from the barley *Mla *gene that were used for screening the library were described earlier (Mago *et al*. 2004). For high-throughput isolation of DNA from selected BAC clones, the alkaline lysis protocol was adapted for 96-well plate format. BAC clones were grown overnight in 300 μl LB with chloramphenicol (12.5 μg/ml) in 96-well plates. Cells were harvested by centrifugation at 2,000 rpm for 20 min. The cells were resuspended in 50 μl GTE with RNaseA and lysozyme. 100 μl of lysis buffer was added and mixed by gentle vortexing followed by addition of 75 μl of chilled potassium acetate and mixed. The plate was centrifuged for 30 min at 3,000 rpm. 200 μl of supernatant was transferred to a fresh plate and DNA precipitated by using 0.6 volume of cold isopropanol. After centrifugation for 30 min at 3,000 rpm, the DNA was washed with 70% ethanol, air dried and resuspended in 25 μl sterile water. The DNA was used directly for restriction digestion. For individual BAC clones, DNA was isolated using the modified alkaline lysis protocol (Sinnett *et al*. 1998).

## Authors' contributions

HŠ, JŠ and JJ constructed the 1RS BAC library and drafted the manuscript. JB screened the library with chromosome-specific markers. PK and PS performed flow cytometric analyses and sorting. JČ prepared samples for flow cytometry. MK performed FISH on flow-sorted fractions. RM used the library to isolate *SrR *gene. TL selected cytologically the parents providing the seeds for sorting and together with JD conceived and supervised the project. JD prepared a final version of the manuscript. All authors read and approved the final manuscript.
